# Comparison of staged lateral lumbar interbody fusion combined two-stage posterior screw fixation and two osteotomy strategies for adult degeneration scoliosis: a retrospective comparative study

**DOI:** 10.1186/s12891-023-06449-z

**Published:** 2023-05-16

**Authors:** Dingli Xu, Kaifeng Gan, Xuchen Zhao, Leidong Lian, Xudong Hu, Ni Luo, Weihu Ma

**Affiliations:** 1 Health Science Center, Ningbo University Zhejiang, Ningbo, China; 2 Orthopedic Department, The Affiliated Lihuili Hospital of Ningbo University, Ningbo, China; 3 Orthopedic Department, Ningbo No.6 Hospital, Zhejiang, Ningbo China

**Keywords:** Adult degeneration scoliosis, Lateral lumbar interbody fusion, Pedicle subtraction osteotomy, Ponte osteotomy

## Abstract

**Aims:**

The commonly used treatments of adult degeneration scoliosis (ADS) were posterior long segment screw fixation with osteotomies. Recently, lateral lumbar intervertebral fusion combined two-stage posterior screw fixation (LLIF + PSF) as a new strategy without osteotomy. Herein, this study aimed to compare the clinical and radiological outcomes among LLIF + PSF and pedicle subtraction osteotomy (PSO), posterior column osteotomies (PCO).

**Methods:**

Totals of 139 ADS patients underwent operation with 2 years longer follow-up visit between January 2013 and January 2018 in Ningbo No.6 Hospital were enrolled into this study. 58 patients were included in PSO group, 45 in PCO group and 36 in LLIF + PSF group, The clinical and radiological data were reviewed from medical records. Baseline characteristic, perioperative radiological data (sagittal vertical axis (SVA), coronal balance (CB), Cobb angle of Mian curve (MC), Lumbar lordosis (LL), pelvic tilt (PT) and pelvic incidence-lumbar lordosis mismatch (PI-LL)), clinical outcomes (VAS of back and leg, Oswestry disability index (ODI) and Scoliosis Research Society 22-question Questionnaire (SRS-22)) and complications were evaluated and compared.

**Result:**

There were no significantly difference in baseline characteristics, preoperative radiological parameters and clinical outcomes among three groups. LLIF + PSF group was significantly shorter in operation time than other two groups (P < 0.05), whereas significant longer hospital stay was observed in LLIF + PSF group (P < 0.05). As for radiological parameters, LLIF + PSF group had significantly improvement in SVA, CB, MC, LL and PI-LL (P < 0.05). Moreover, LLIF + PSF group achieved significantly less correction loss in SVA, CB and PT than PSO and PCO group (1.5 ± 0.7 VS 2.0 ± 0.9 VS 2.2 ± 0.8, P < 0.05; 1.0 ± 0.4 VS 1.3 ± 0.5 VS 1.1 ± 0.7, P < 0.05 and 4.2 ± 2.8 VS 7.2 ± 3.1 VS 6.0 ± 2.8, P < 0.05). Significantly recovery in VAS of back and leg, ODI score and SRS-22 were found among all groups, however, LLIF + PSF shown significant better clinical therapy maintain at follow-up visit than other two groups (P < 0.05). There were no significantly difference in complications among groups (P = 0.66).

**Conclusion:**

Lateral lumbar interbody fusion combined two-stage posterior screw fixation (LLIF + PSF) can achieve comparable clinical therapy for adult degeneration scoliosis as osteotomy strategies. However, furthermore more studies need be taken for verifying the effect of LLIF + PSF in the future.

## Introduction

The prevalence of adult degenerative scoliosis is increasing throughout the aging process, which has a debilitating effect on people’s health. Adult degenerative scoliosis (ADS) is a three-dimensional spine deformity in skeletally mature adults with a Cobb angle > 10° in the coronal plane [1]. According to Diebo et al., ADS is a chronic condition caused by bone and soft tissue degeneration. The degeneration process begins in the disc anatomy and biochemical and biomechanical properties of the intervertebral disc. This leads to pathological changes in the load-bearing unit and vertebral structure remodeling, and facet joint instability [2]. Presentations such as cosmetic deformity, back and leg pain, disability, and neurological complaints and related medical treatment greatly burden on social, economic, and mental health [3].

Decompression of the involved neural element; realignment of the global spine balance in coronal and sagittal planes; and minimization of perioperative risks, complications, and reoperation were the key aims of surgical treatments for ADS [4]. Decompression and short segment fusion for proximal junctional kyphosis (PJK), internal fixation failure, iatrogenic postoperative instability, and even the need for reoperation are possible consequences of subsequent scoliosis progression and recurrent radicular pain caused by foraminal stenosis [5–7]. Consequently, surgeons tended to prefer long segment fusion such as osteotomies like posterior column osteotomies (PCO), pedicle subtraction osteotomies (PSO), and posterior vertebral column resections (PVCR), because long segment fixation is often necessary due to avoid stopping the fixation at the apex of Thoracic kyphosis and osteotomy is needed to correct the spinal alignment to the ideal spinopelvic parameters. As time passed, several osteotomies deficiencies were reported. In a retrospective study, Bourghli et al. found that PSO significantly improved the sagittal vertical axis (SVA) (from 130.62 ± 63 mm to 43.57 ± 28.6 mm), PT (from 31.02 ± 10° to 21.91 ± 8.5°), and PI-LL (from 30.05 ± 15° to 6.1 ± 9.3°) in 102 patients with ADS. However, 23 (22.5%) of 103 patients required revision for PJK, pseudarthrosis, deep surgical infection, epidural hematoma, and neurological deficit [8]. Similarly, Penalosa et al. reported that early instrumentation failure had occurred in 9 of 46 patients treated with PSO [9].

Lateral lumbar interbody fusions have been more common during the last decade, making surgeons more aware of their potential for correcting deformities. In a retrospective study from 2008 to 2018, Passias et al. collected and compared the characteristic of 752 adult patients with spinal deformities who had surgical treatments and found that three-column osteotomies were rarely used, even in cases of severe deformity (SVA > 9.5 cm). And the reason is that in last decades lateral intervertebral fusion have been used for preventing PJK, which play more role to the ASD corrective surgery [10]. Moreover, Strom et al. reported 32 adult spinal deformity patients treated with Lateral interbody fusion combined with open posterior surgery achieved significant improvement in VAS of back, ODI and less blood loss than patients who treated with PSO (P < 0.05) [11]. Although decreasing blood loss and minimizing recovery time are two reasons lateral lumbar interbody fusion with posterior long segment screw fusion has gained popularity for correcting spine deformities, some researchers have proposed that LLIF + PSF cannot directly decompress the spinal cord and spinal nerve root. Thus, this technique should be applied to a subset of patients with ADS who still have compensating mechanisms [12].

ADS has become more prevalent with the aging progress. However, there is still controversy about the clinical outcome of LLIF + PSF for treating ADS, and there is a lack of literature that compares LLIF + PSF to osteotomy surgeries such as PSO or PCO in terms of radiological correction and health-related quality-of-life outcomes. This study aimed to compare the clinical therapies of LLIF + PSF, PSO, and PCO for adult degenerative scoliosis and to assess the safety of LLIF + PSF.

## Methods

### Patients

In total, 139 patients with adult degenerative scoliosis who underwent surgery using one of three different methods—LLIF + PSF, PSO, and PCO—were enrolled in our study. These patient’s clinical and radiological outcomes were obtained from their medical records at Ningbo No. 6 Hospital between January 2013 and January 2018. The inclusion criteria were: (1) Diagnosed as adult degenerative scoliosis by coronal plane Cobb angle > 10º in X-ray; (2) Clinically presented about low back pain and leg symptoms; (3) The parameters in the anteroposterior and lateral spine full-length X-ray met the criteria of Lenke-Silva level V or VI (Cobby > 30 º, olisthesis > 2 mm, with lumbar kyphosis) [4]; (4) With a minimum of 2 years follow-up visit; (5) They were treated with long-segment fixation. The exclusive criteria were: (1) Patients with ADS secondary to other diseases, such as tuberculosis, a fracture, a tumor, and Kummell disease; (2) Patients with a history of spine surgery; (3) Patients with congenital spine abnormalities, such as hemivertebra, congenital block vertebrae, and butterfly vertebrae.

All procedures involving human subjects followed the institution’s ethical standards, the 1964 Helsinki Declaration, and any subsequent amendments or comparable ethical standards. The Research and Ethics Committee of Ningbo No. 6 Hospital (No. 20,210,023) approved this study.

## Surgery procedure

### LLIF + PSF

Patients underwent a two-step surgical procedure, with the first stage including LLIF and the second stage involving posterior screw fixation one week later. For the first stage of LLIF, the patients have positioned in the lateral decubitus position on an appropriately flexible operating table while being monitored electromyographically. Approaching the concave side of the spine, the bridging osteophyte, annulus fibrosus, and anterior longitudinal ligament were cleared accordingly. After gradually expanding dilators to release contracture tissue, a tubular retractor should be placed using the transpsoas approach. A discectomy and annulus release on the opposite side was also performed. Finally, allograft was used to implant a suitable size polyether ether ketone interbody device. Anteroposterior and lateral spine full-length radiographs were used to plan the second stage of posterior screw fixation one week after the patients’ initial evaluation. Precisely, after soft tissue exposed, interspinous ligament resection and multilevel Schwab Grade I facetectomy were performed for decompression. Then segmental pedicle screws and autogenous iliac bolts were placed routinely.

**Fig. 1:**
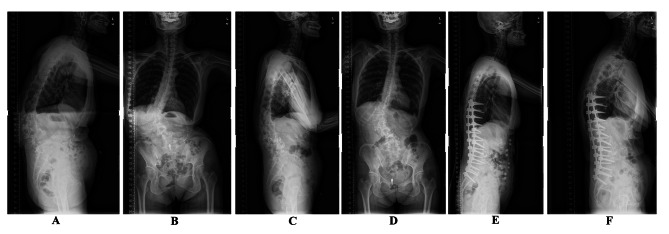
A 64-year-old female patient with low back pain treated with lateral lumbar interbody fusion combine two-stage posterior screw fixation. (**A**, **B**) A preoperative standing anteroposterior and lateral full-length spine X-ray showed the following findings: Cobb angle of lumbar curve = 58.9°, LL = 4.3°, PT = 31.3°, LL-PI = 35.6°, SVA = 12.0 cm, coronal balance was 9.2 cm. (**C**, **D**) A standing anteroposterior and lateral full-length spine radiograph at postoperatively following ALIF from L2-5. The radiograph revealed Cobb angle of main curve = 48.7°, LL = 11.8°, PT = 21.6°, LL-PI = 24.3°, SVA = 5.9 cm, coronal balance was 8.5 cm. (**E**) After second-stage posterior fixation from T9-S2 combined with PSF, the standing anteroposterior and lateral full-length spine radiographs showed the Cobb angle of main curve = 23.2°, LL = 35.4°, PT = 9.6°, LL-PI = 8.5°, SVA = 3.8 cm, coronal balance was 1.2 cm. (**F**) At final follow-up the standing anteroposterior and lateral full-length radiographs showed the Cobb angle of main curve = 24.6°, LL = 32.9°, PT = 10.9°, LL-PI = 9.5°, SVA = 4.8 cm

### PSO group

**Fig. 2:**
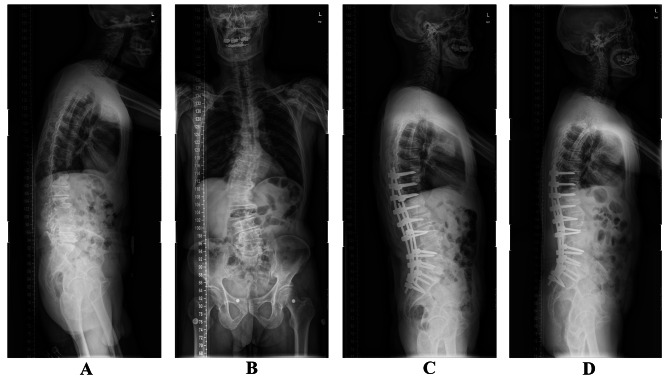
A 75-year-old male patient with low back and leg pain treated with PSO at L3 assised with satellite rods. (**A**, **B**) A preoperative standing anteroposterior and lateral spine full-length X-ray: Cobb angle of main curve = 45.2°, LL = 10.2°, PT = 44.2°, LL-PI = 30.6°, SVA = 6.5 cm, coronal balance was 4.1 cm. (**C**) A standing lateral full-length spine radiograph at postoperative. The radiograph showed Cobb angle of main curve = 24.9°, LL = 32.1°, PT = 14.5°, LL-PI = 3.7°, SVA = 0.4 cm, coronal balance was 0.9 cm. The patient’s low back and leg pain was completely disappeared. (**D**) At final follow-up the standing anteroposterior and lateral full-length radiographs showed the Cobb angle of main curve = 24.6°, LL = 30.9°, PT = 16.2°, LL-PI = 6.8°, SVA = 0.5 cm

Patients were placed in a prone position in the flexed operating bed after receiving anesthesia, with a mattress under their forehead, chest, and abdomen to ensure that the abdomen hung freely. The posterior elements were exposed through a midline skin incision. First, pedicle screws were inserted, and in case of an abrupt sagittal translation during PSO, temporary pre-contoured rods were also inserted. Then an extended central laminectomy and a transverse process were performed. Once the posterior vertebral wall had been sufficiently thinned, it was removed. Later, a high-speed drill was used to resect the lateral vertebral wall. Finally, closing-opening wedge osteotomy was performed by fracture of the anterior vertebral cortex. The lamina was preserved to serve as the fusion bed for the remaining adjacent lamina of the osteotomized vertebra. Fixation with pre-contoured rods and straightening of the flexed operating table caused trunk extension and improved overall imbalance and spine stability (Fig. 2).

### PCO group

Patients were placed in a prone position in the flexed operating bed after receiving anesthesia, with a mattress under their forehead, chest, and abdomen to ensure that the abdomen hung freely. Pedicle screws and temporary pre-contoured rods were inserted after the posterior elements were exposed. Removing superior and inferior articular processes bilaterally and interlaminar spaces resulted in multi-level wedge osteotomies. Pre-contoured rods were tightened after the spinous process, and facet joints were used as autografts for correction. The osteotomy is completed when segmental spine mobility is confirmed, and neuromonitoring remains unchanged. Finally, the wound was closed over two suction drains.

All patients were treated in an intensive care unit for 1 to 2 days after surgery. Three months after surgery, patients could ambulate using a molded thoracic-lumbosacral orthosis.

### Outcome evaluation

Age, gender, body mass index (BMI), operation time, hospital stay, and fusion level were the baseline characteristics of all patients.

**Fig. 3:**
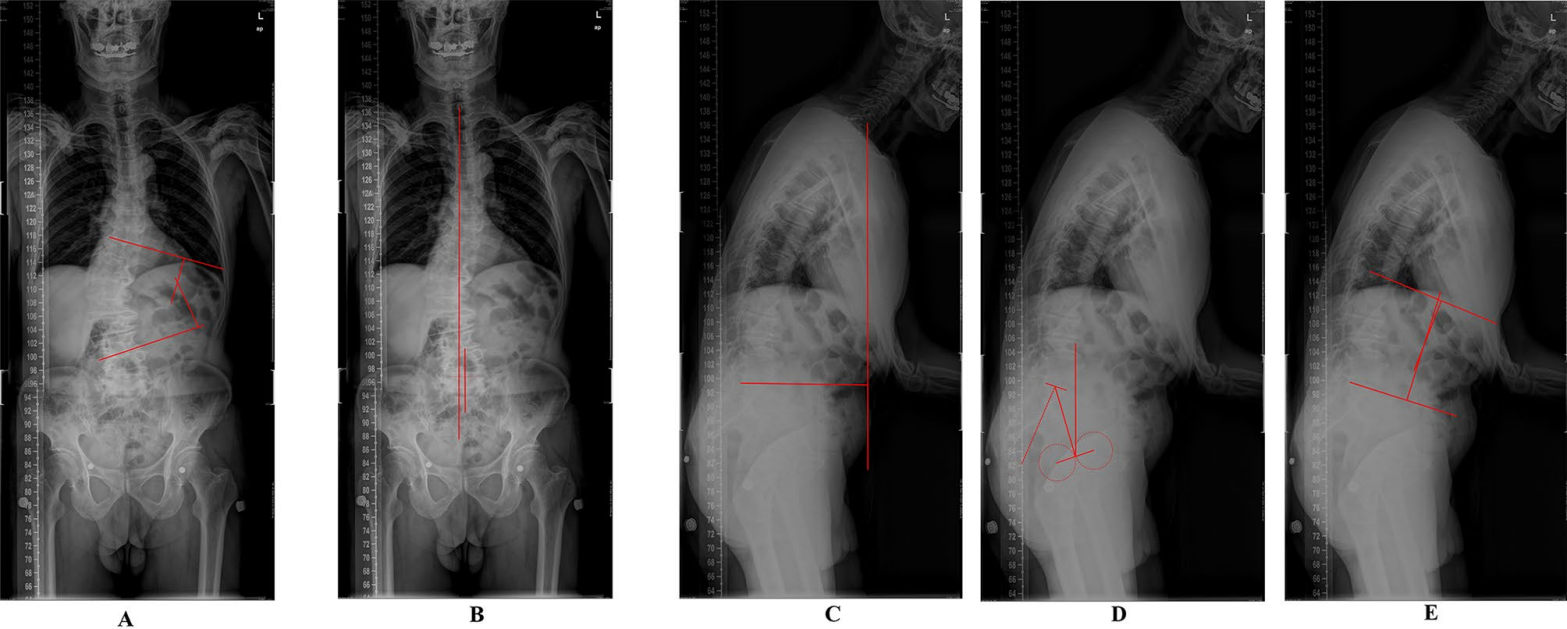
The measurement of radiological parameters. Cobb angle of main curve (MC), coronal balance (CB), sagittal vertical axis (SVA), pelvic tilt (PT), lumber lordosis (LL) and pelvic incidence (PI). **A**. Cobb angle of main curve (MC): the greatest cobb angle at main curve of the spine, measured from the upper endplate of a upper vertebra to the lower endplate of a lower vertebra; **B**. Coronal balance (CB): the horizontal distance between C7 plumb line and center sacral vertical line; **C**. Sagittal vertical axis (SVA): the vertical distance between C7 plumb line and the upper back corner of the S1 endplate; **D**. Pelvis tilt (PT) and: the angle between lines originating at the bicoxofemoral axis and extending vertically and to the middle of the superior endplate of S1, pelvic incidence (PI): angle between a line perpendicular to the superior endplate of S1 and the line connecting the superior endplate of S1 to the bicoxofemoral axis; **E**. lumber lordosis (LL): the Cobb angle between the upper endplate of L1 and the upper endplate of S1;

Radiological outcomes, including the main Cobb angle (MC), coronal balance (CB), SVA, and spinopelvic parameters, were measured by two experienced radiologists, and the details are as follows [13]. MC: the Cobb angle of the major curve; CB: the horizontal distance between the C7 plumb line and the center sacral vertical line; SVA: the vertical distance between the C7 plumb line and the upper back corner of the S1 endplate; LL: the Cobb angle between the upper endplate of L1 and the upper endplate of S1; PT: the angle between lines originating at the bicoxofemoral axis and extending vertically and to the middle of the superior endplate of S1; and PI: the angle formed between a line perpendicular to the superior endplate of S1 and the line connecting the superior endplate of S1 to the bicoxofemoral axis (Fig. 3).

As for clinical outcomes, the pain in the back and leg was assessed using a visual analog scale (0: no pain, 10: most severe pain) for the back (VAS of back) and the leg (VAS of the leg), respectively. Oswestry disability index (ODI) and the Scoliosis Research Society 22-question Questionnaire (SRS-22) were used to evaluate the daily living abilities of patients [14]. Meanwhile, the complications were also collected.

### Statistical analysis

Statistical Package for Social Sciences 24.0 (IBM Corp., Armonk, New York, USA) was used for the statistical analysis. All data were expressed as mean ± standard deviation. The Shapiro-Wilk test was used to determine the normality of continuous data. Age, BMI, ODI, and SRS-22 measurement data were compared using analysis of variance. The categorical data were compared using the χ2 test. P < 0.05 was considered statistically significant.

## Result

### Baseline outcomes

We enrolled 139 eligible patients in our study, including 68 males and 71 females, and retrospectively reviewed their medical records. There were no significant differences in age, gender, BMI, and fusion level (P > 0.05) among the 58 patients who underwent PCO, and 36 underwent LLIF + PSF. Additionally, there was a significantly reduced blood loss in the LLIF + PSF group (469.4 ± 109.1 mL vs. 912.1 ± 137.7 mL vs. 733.3 ± 110.8 mL, P < 0.05) than in the PSO group (5.6 ± 0.4) h and PCO group (6.3 ± 0.5) h after operation time of LLIF + PSF group (4.6 ± 0.3) h (P < 0.05). The LLIF + PSF group needs a significantly longer hospital stay than the other two groups (P < 0.05). Table 1 displays details of the baseline characteristics.

**Table 1:** The comparison of baseline characteristics among three groups

	PSO	PCO	LLIF + PSF	P value
Number	58	45	36	
Age(yr)	61.8 ± 7.2	62.7 ± 7.8	63.6 ± 7.3	0.52
Gender				
Male	29	23	16	0.82
Female	29	22	20	
BMI(Kg/m2)	26.7 ± 1.8	27.3 ± 2.0	26.5 ± 1.6	0.14
Blood loss (ml)	912.1 ± 137.7	733.3 ± 110.8	469.4 ± 109.1	< 0.001^φ^
Operation time(H)	5.6 ± 0.4	6.3 ± 0.5	4.6 ± 0.3	< 0.001^φ^
Hospital stay(D)	7.3 ± 1.7	7.4 ± 1.8	13.9 ± 1.4	< 0.001^φ^
Fusion level	10.4 ± 1.1	10.3 ± 1.1	10.6 ± 1.2	0.51
SVA(cm)	9.6 ± 1.9	9.5 ± 2.0	10.2 ± 1.5	0.17

φ: p < 0.05

### Clinical outcomes

Preoperatively, there were no significant differences in VAS of the back, VAS of the leg, ODI, and SRS-22 among the three groups (P > 0.05). After surgery, the LLIF + PSF group improved significantly in back pain relief, ODI, and SRS-22 at a 2-year follow-up visit (P < 0.05). Precisely, at the 2-year follow-up visit, the VAS of the back in the LLIF + PSF group (1.1 ± 0.8) was significantly lower than that of the other two groups (2.0 ± 0.8 and 1.4 ± 1.0) (P < 0.05). At the final follow-up, patients in the LLIF + PSF group had significantly higher ODI scores (14.7 ± 3.3) than those in the PSO and PCO groups (16.7 ± 2.2 and 16.6 ± 2.1) (P < 0.05). Moreover, the SRS-22 significantly improved the LLIF + PSF group more than the PSO and the PCO groups (P < 0.05). Showed in Table 2.

**Table 2:** The comparison of clinical outcomes among three groups

	PSO	PCO	LLIF + PSF	P value
VAS of back				
Preoperative	5.6 ± 1.2	5.2 ± 1.1	5.6 ± 1.2	0.11
Postoperative	3.1 ± 0.8	2.8 ± 1.1	3.1 ± 0.9	0.23
1 year follow-up	2.5 ± 0.5	1.8 ± 0.9	2.7 ± 0.9	< 0.05^φ^
2 years follow-up	2.0 ± 0.8	1.4 ± 1.0	1.1 ± 0.8	< 0.05^φ^
VAS of leg				
Preoperative	4.1 ± 0.9	4.3 ± 0.8	4.0 ± 0.8	0.34
Postoperative	2.2 ± 0.9	2.1 ± 0.9	2.1 ± 0.8	0.94
1 year follow-up	1.7 ± 0.8	1.6 ± 0.7	1.7 ± 0.7	0.86
2 years follow-up	1.3 ± 1.0	0.9 ± 0.8	1.0 ± 0.7	0.07
ODI				
Preoperative	45.0 ± 7.7	42.9 ± 6.0	43.8 ± 9.4	0.42
Postoperative	20.2 ± 2.9	18.9 ± 3.3	20.1 ± 2.9	0.1
1 year follow-up	18.6 ± 4.2	17.3 ± 3.0	19.8 ± 3.1	< 0.05^φ^
2 years follow-up	16.7 ± 2.2	16.6 ± 2.1	14.7 ± 3.3	< 0.05^φ^
SRS-22				
Preoperative	1.8 ± 0.7	2.0 ± 0.7	2.1 ± 0.7	0.13
Postoperative	3.2 ± 0.7	3.3 ± 0.6	3.4 ± 0.7	0.23
1 year follow-up	4.0 ± 0.8	4.0 ± 0.7	4.1 ± 0.6	0.74
2 years follow-up	4.2 ± 0.9	4.3 ± 0.7	4.7 ± 0.6	< 0.05^φ^
Complications	9	2	1	< 0.05^φ^

φ: p < 0.05

### Radiological outcomes

The spinopelvic parameters were similar in all three preoperatively. However, patients in the LLIF + PSF group showed significantly better improvement after surgery and lower correction loss at the final follow-up visit (P < 0.05). Patients in the LLIF + PSF group had significantly better SVA than those in the other two groups (5.5 ± 0.6 vs. 6.6 ± 0.7 vs. 6.2 ± 0.5, P < 0.05), and the lowest correction loss was observed from the postoperative to the final follow-up period (1.5 ± 0.7 vs. 2.0 ± 0.9 vs. 2.2 ± 0.8, P < 0.05). The outcomes of PI-LL, PT, and coronal balance improvement and correction were similar to those of SVA. Regarding the Cobb angle of the main curve and lumber lordosis, the LLIF + PSF group significantly improved (P < 0.05). However, there was no significant difference in corrective loss among groups at the follow-up visit (P > 0.05). In Table 3, the details of the radiological outcomes are shown.

**Table 3:** The comparison of radiological outcomes among three groups

	PSO	PCO	LLIF + PSF	P value
SVA(cm)				
Preoperative	9.6 ± 1.9	9.5 ± 2.0	10.2 ± 1.5	0.17
Postoperative	4.5 ± 0.9	4.0 ± 1.0	4.0 ± 0.4	< 0.05^φ^
1 year follow-up	5.5 ± 0.9	5.6 ± 1.0	4.5 ± 0.3	< 0.05^φ^
2 years follow-up	6.6 ± 0.7	6.2 ± 0.5	5.5 ± 0.6	< 0.05^φ^
Correction loss	2.0 ± 0.9	2.2 ± 0.8	1.5 ± 0.7	< 0.05^φ^
coronal balance (cm)				
Preoperative	4.3 ± 0.5	4.5 ± 0.6	4.3 ± 0.4	0.18
Postoperative	1.7 ± 0.6	1.4 ± 0.4	1.1 ± 0.5	< 0.05^φ^
1 year follow-up	2.3 ± 0.5	1.8 ± 0.4	1.7 ± 0.5	< 0.05^φ^
2 years follow-up	3.1 ± 0.4	2.5 ± 0.6	2.2 ± 0.6	< 0.05^φ^
Correction loss	1.3 ± 0.5	1.1 ± 0.7	1.0 ± 0.4	< 0.05^φ^
Main curve(°)				
Preoperative	59.5 ± 8.1	57.0 ± 10.0	57.6 ± 11.8	0.38
Postoperative	23.4 ± 3.0	21.8 ± 2.6	18.3 ± 3.5	< 0.05^φ^
1 year follow-up	28.4 ± 4.7	27.4 ± 5.4	25.6 ± 5.2	< 0.05^φ^
2 years follow-up	31.9 ± 3.5	31.5 ± 3.8	27.6 ± 4.7	< 0.05^φ^
Correction loss	8.5 ± 4.2	9.7 ± 4.1	9.3 ± 6.3	0.39
Lumber lordosis(°)				
Preoperative	13.0 ± 5.3	11.8 ± 4.4	12.6 ± 4.7	0.47
Postoperative	41.8 ± 3.6	42.6 ± 4.0	44.8 ± 4.6	< 0.05^φ^
1 year follow-up	35.5 ± 3.0	34.4 ± 2.9	39.4 ± 5.3	< 0.05^φ^
2 years follow-up	33.6 ± 2.8	33.7 ± 3.2	37.9 ± 3.5	< 0.05^φ^
Correction loss	8.2 ± 4.2	7.7 ± 5.3	6.8 ± 3.2	0.34
PI-LL(°)				
Preoperative	33.1 ± 6.4	33.2 ± 5.5	34.3 ± 7.7	0.65
Postoperative	6.1 ± 2.6	4.8 ± 2.9	4.0 ± 2.4	< 0.05^φ^
1 year follow-up	7.9 ± 2.1	8.2 ± 1.9	5.2 ± 2.2	< 0.05^φ^
2 years follow-up	11.6 ± 2.0	10.4 ± 2.0	7.3 ± 1.8	< 0.05^φ^
Correction loss	5.5 ± 3.2	5.6 ± 3.8	3.3 ± 1.8	< 0.05^φ^
PT(°)				
Preoperative	35.0 ± 5.8	34.4 ± 4.3	33.8 ± 3.1	0.46
Postoperative	12.1 ± 2.4	13.0 ± 2.2	12.0 ± 2.6	0.11
1 year follow-up	15.2 ± 2.1	16.9 ± 2.5	14.2 ± 2.6	< 0.05^φ^
2 years follow-up	19.3 ± 2.5	19.0 ± 2.1	16.2 ± 2.4	< 0.05^φ^
Correction loss	7.2 ± 3.1	6.0 ± 2.8	4.2 ± 2.8	< 0.05^φ^

φ: p < 0.05; Correction loss: The difference between 2 years follow-up and postoperative

### Complication

As for complications, a total of 9 complications were observed in the PSO group, 2 in the PCO group, and 1 in the LLIF + PSF group, with a significant difference among the three groups (P < 0.05). Two patients in the PSO group had deep wound infections; one was treated with antibiotic therapy, and the other 7 had failed conservative treatment and received wound washouts. Five cases of wound exudation and two cases of disc wedging progression were effectively treated with conservative treatments. In the PCO group, there was a case of wound exudation, and one case had proximal junctional kyphosis that was treated conservatively. One patient in the LLIF + PSF group had radiographic adjacent segmental degeneration which was treated successfully managed by conservative treatment.

## Discussion

### The pathophysiology and related treatments of ADS

Asymmetric degeneration of the intervertebral discs and facet joints causes adult degenerative lumbar scoliosis, a 3-dimensional deformity with coronal Cobb angle > 10º [15]. Degenerative alterations in the anatomy and biochemistry of the discs and facet joints, such as reduced disc height, loss of water and proteoglycan content, and increased enzymatic degradation, lead to an imbalance in the spine’s load-bearing, which in turn causes bone remodeling and facet joint instability. Spine deformity results from this cycle of remodeled bones and progressing spine degeneration [16]. Degenerative changes to bones and soft tissues, such as spondylolisthesis and rotatory subluxation, frequently bring on back pain and radiculopathy. Patients with only mechanical back pain or absence of significant stenotic or radiculopathy can get nonoperative treatment for ADS; however, most of these patients will eventually require surgery due to progressive spine degeneration. Patients in categories V or VI of the Lenke-Sliva classification should receive long-segment fixation and decompression, and osteotomies were also performed to correct curvature and imbalance [4]. Schwab et al. proposed an osteotomy classification system with six grades corresponding to an increased risk of instability [17].

Recently, the most popular osteotomy strategies in clinical practice were PCO, PSO, and PVCR. The original PCO concept, the Smith Petersen osteotomy, involved posterior element resection and posterior column compression; however, there was a high risk of vascular injury anterior to the spine and disruption of the anterior longitudinal ligament. Ponte et al. reported a modified method of Smith-Petersen osteotomy that can correct deformity without disrupting the anterior longitudinal ligament because the center of rotation of the Ponte osteotomy depends on the posterior disc annulus [18]. PCOs are frequently used to treat spine deformities, particularly when sagittal correction (posterior column shortening) is required [19]. In a multicenter prospective study, Buckland et al. reported that only 6 out of 1611 Ponte osteotomy patients experienced neurological complications [20]. Moreover, Korovessis et al. reported that 67 elderly patients with adult spinal deformities received multiple Ponte osteotomies, and they significantly improved their radiological parameters (LL, PI-LL, and SVA) and clinical outcomes (ODI and SF-36) [21].

Thomasen et al. proposed pedicle subtraction osteotomy in 1985. It was described as a transpedicular V-shaped wedge three-column osteotomy [22]. The “eggshell” procedure, which uses a posterior transpedicular approach to achieve anterior decompression and posterior fusion with an average curve correction of 26º, is a recent advancement in the surgical technique for PSO [23]. PSO achieved a stable correction by shortening the middle and posterior columns while leaving the anterior column unaffected by anterior longitudinal ligament disruption. In contrast, PSO increased complications, operation time, and blood loss. Passias et al. reported that 20 patients with lumbar spine deformity underwent PSO and experienced significant improvement in SVA (preoperation 169.1 ± 89.1 mm vs. final follow-up 53.2 ± 19.1 mm, P < 0.05), PT (preoperation 39.1 ± 13.4º vs. final follow-up 24.8 ± 9.6 º, P < 0.05), and ODI (preoperation 32.9 ± 10.1 vs. final follow-up 16.1 ± 6.4, P < 0.05), whereas 3 out of 20 (15%) patients experienced complications, such as an intraoperative dural tear combined with postoperative parietal aeration, cerebrospinal fluid leakage, incision delay healing, and internal fixation break [24].

### The necessity of LLIF + PSF

There are high risks associated with osteotomy surgery, such as pseudoarthrosis, hardware breakage, increased blood loss, and proximal junctional kyphosis [25]. According to Hyun et al., 13 patients underwent pedicle subtraction osteotomy, and 16 complications were observed, such as massive bleeding (> 5000 ml), dural tears, craniospinal fluid leakage, rod breaks, and kyphosis progression with collapse [26]. Lateral lumbar intervertebral fusion with stage posterior long segment screw fixation has become a non-osteotomy method of correcting curves and imbalances. Lumbar intervertebral fusion can indirectly relieve nerve root compression by increasing intervertebral disc height and correcting the right curve with circumferential fusion. Meanwhile, minimally invasive surgery, lateral lumber intervertebral fusion, and posterior screw fixation can correct deformity using osteotomy, decrease blood loss, and shorten recovery times [27–29]. According to Wu et al., pedicle screw fixation and lumbar interbody fusion were performed on 26 ADS patients. Their Cobb angle of the lumbar curve, lumber lordosis, and ODI score all significantly improved (P < 0.05) [30]. Similarly, Katz et al. analyzed 27 patients who underwent a lateral lumbar interbody fusion with posterior instrumentation for degenerative scoliosis. They found that the Cobb angles of patients significantly improved (from 21.1º to 7.9º, P < 0.05), and their SF-12 and ODI scores also significantly improved and were maintained at a follow-up visit (P < 0.05). Moreover, shorter operating times (178–236 min) and less operating blood loss (100–202 mL) were also observed [31].

### The effectiveness of LLIF + PSF

Compared to the PSO and PCO group during the follow-up visit, the LLIF + PSF group significantly improved and maintained clinical results, including VAS of back, ODI, and SRS-22 (P < 0.05). Additionally, when compared to the other two groups, the operation blood loss (469.4 ± 109.1 mL vs. 912.1 ± 137.7 mL vs. 733.3 ± 110.8 mL, P < 0.05), operation time (4.6 ± 0.3 h vs. 5.6 ± 0.4 vs. 6.3 ± 0.5 h, P < 0.05) and complications were all much reduced in LLIF + PSF group. The blood loss of PSO is roughly twice as much as LLIF + PSF (2910 VA 1466 ml, P < 0.01), and posterior operative complications were also significantly higher in the PSO group (P < 0.05), according to Leveque et al. retrospective analysis of the medical records of 14 ADS patients with PSO and 13 patients treated with LLIF with posterior screw fixation [32]. In a similar vein, Wang et al. reported that in 23 patients with ADS who underwent LLIF + PSO funding, the average blood loss was 477 ml [33]. Less blood loss and complication rates may be attributable to the following factors: (1) PSO and PCO osteotomy strategies increase cancellous bone bleeding; (2) PSO corrects deformity by single-segment osteotomy, which increases the risk of hardware failure and proximal junctional kyphosis due to non-harmonious realignment [34].

Regarding radiological results, all patients show significantly improved SVA, coronal balance, Cobb angle of the main curve, LL, PT, and PI-LL values (P < 0.05). And when compared to the PSO and PCO group, the LLIF + PSF group demonstrated a considerably less corrective loss in the following areas: SVA (1.5 ± 0.7 cm vs. 2.2 ± 0.8 cm vs. 2.0 ± 0.9 cm, P < 0.05), CB (1.0 ± 0.4º vs. 1.1 ± 0.7º vs. 1.3 ± 0.5º, P < 0.05), PT (3.3 ± 1.8º vs. 5.6 ± 3.8º vs. 5.5 ± 3.2º, P < 0.05), and PI-LL (4.2 ± 2.8º vs. 6.0 ± 2.8º vs. 7.2 ± 3.1º, P < 0.05). A total of 26 patients diagnosed with adult degeneration scoliosis and treated with LLIF + PSF were reported to have similar outcomes by Tempel et al. The clinical results were improved and maintained at final follow-up (P < 0.05), while the mean Cobb angle of the main curve significantly decreased after the operation and maintained at final follow-up (preoperative 41.1º vs. postoperative 26.0º vs. final follow-up 29.4º, P < 0.01) [35]. According to the hypothesis by Le et al., posterior screw fixation combined with LLIF can considerably increase segmental lordosis (13.02 ± 8.37º vs. 15.30 ± 7.84º, P < 0.001) and disc heights (6.51 ± 2.49 mm vs. 10.08 ± 2.68 mm, P < 0.001) than preoperative levels [36]. Additionally, Li et al. proposed that first-stage LLIF could change Lenke-Silva classification and determine the ideal fusion level in second-stage surgery that can avoid osteotomy in Lenke-Silva V and VI patients. They found that 88% of patients had their Lenke-Silva classification changed, and significant improvement and well-maintained spinopelvic parameters like PI-LL, PT, and SVA were seen [37].

The benefits of LLIF + PSF include (1) releasing soft tissue tension and increasing disc height, which allows for better deformity correction and rendered support in the anterior and middle column than PSO and PCO; (2) changing the Lenke-Silva classification by performing a first LLIF, which can prevent osteotomies and determine the optimal fusion level in second operation; and (3) reducing operation time, blood loss, and postoperative complications in comparison to osteotomy strategies like PSO and PCO [38–40].

## Limitations

This study has some limitations. First, because this is a retrospective, single-center study vulnerable to biases, all eligible patients were identified by inclusion and exclusion criteria to minimize biases. Second, different surgeons conducted those surgeries over five years, and the cumulative experience of the surgeons may have some influence on the outcomes.

## Conclusion

In conclusion, LLIF + PSF can release soft tissue and increase disc height, allowing better deformity correction and improved support in the anterior and middle column, decreased operation time, blood loss, and postoperative complications compared to osteotomy PSO and PCO. LLIF + PSF may be an effective and feasible strategy for patients with ADS to yield comparable clinical and radiological outcomes as osteotomy methods.

## Data Availability

The data that support this study are available from the corresponding authors upon request.
